# Advanced Cooling Textiles: Mechanisms, Applications, and Perspectives

**DOI:** 10.1002/advs.202305228

**Published:** 2023-12-22

**Authors:** Xueping Zhang, Fei Wang, Hanyu Guo, Fengqiang Sun, Xiangshun Li, Chentian Zhang, Chongwen Yu, Xiaohong Qin

**Affiliations:** ^1^ Key Laboratory of Textile Science & Technology Ministry of Education College of Textiles Donghua University Shanghai 201620 China; ^2^ State Key Laboratory for Modification of Chemical Fibers and Polymer Materials College of Materials Science and Engineering Donghua University Shanghai 201620 China; ^3^ Key Laboratory of Science & Technology of Eco‐Textile Ministry of Education College of Textiles Donghua University Shanghai 201620 China

**Keywords:** cooling textiles, energy conservation, thermal‐moisture management

## Abstract

High‐temperature environments pose significant risks to human health and safety. The body's natural ability to regulate temperature becomes overwhelmed under extreme heat, leading to heat stroke, dehydration, and even death. Therefore, the development of effective personal thermal‐moisture management systems is crucial for maintaining human well‐being. In recent years, significant advancements have been witnessed in the field of textile‐based cooling systems, which utilize innovative materials and strategies to achieve effective cooling under different environments. This review aims to provide an overview of the current progress in textile‐based personal cooling systems, mainly focusing on the classification, mechanisms, and fabrication techniques. Furthermore, the challenges and potential application scenarios are highlighted, providing valuable insights for further advancements and the eventual industrialization of personal cooling textiles.

## Graphical abstract

This review provides a comprehensive summary of cooling textiles. According to heat transfer mechanisms, cooling textiles can be classified into wearable devices, radiative cooling textiles, thermal conductive cooling textiles, evaporative cooling textiles, and dynamic responsive cooling textiles. The fundamental principles and process routes are emphasized, shedding light on the latest research advancements and future directions in this field.

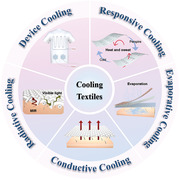

## 1 Introduction

The issue of refrigeration in hyperthermia environments has aroused considerable attention due to its impact on both production and daily life.^[^ ^1^ ^]^ The greenhouse and urban heat island effect leads to the increase in the temperature of buildings and fabrics, which impacts human thermal comfort. The normal operation of human function is highly sensitive to temperature, with an optimal internal temperature of ≈37 °C.^[^ ^2^ ^]^ Temperatures exceeding 40 °C would result in heat stress or even fatal diseases.^[^ ^3^ ^]^ Although the human body has certain automatic thermoregulation mechanisms, such as muscle movement, sweating, and vasodilation, these are usually not enough to achieve thermal comfort in extreme heat environments and intensive human activities.^[^ ^4^ ^]^ To maintain human thermal comfort, building ventilation, and air conditioning systems are widely used to regulate temperature for creating a comfortable indoor environment for a long time. However, the energy consumption of this technology is huge, which not only exacerbates the production of greenhouse gases and global warming but also restricts the application scenarios indoors.^[^ ^5^, ^6^ ^]^ Personal thermal management technology can regulate the heat exchange between the human body and its environment to a certain extent through wearable devices and functional textiles. It helps to maintain a comfortable micro‐climate and improves the ability to cope with the changes in the external environment.^[^ ^7^ ^]^ Additionally, precise and individualized personal thermal management is of great importance for reducing energy consumption and expanding cooling applications.

The human body utilizes various mechanisms to regulate heat dissipation in response to different environmental conditions. There are four main passive heat transfer mechanisms between the body and its surroundings: convection, radiation, conduction, and evaporation.^[^ ^8^ ^]^ The net cooling power (*P_net_*) of the human body can be expressed as^[^ ^9^, ^10^, ^11^, ^12^ ^]^:

(1) $$P_{\mathrm{net}}=P_{\mathrm{rad}}\;-P_{\mathrm{atm}}-P_{\mathrm{solar}}+P_{\mathrm{conv}}+P_{\mathrm{cond}}+P_{\mathrm{evap}}$$

where *P*_rad_, *P*_atm_, *P*_solar_, *P*_conv_, *P*_cond_, and *P*_evap_ are the power densities of thermal radiation emitted by the body, atmospheric radiation absorbed by the body, solar irradiation absorbed by the body, and convective, conductive as well as evaporative power density on the body, respectively. The heat and moisture create a ‘microclimate’ around the skin and clothing. Except for heat, sweat is an essential factor that affects human thermal and moisture comfort.^[^ ^13^ ^]^ Sweat is the body's natural response to regulate its temperature and maintain homeostasis. When the body temperature rises, sweat glands secrete moisture onto the skin's surface, which evaporates to cool down the body. Nevertheless, the evaporation of sweat is hindered in hot and humid microclimates due to the high vapor pressure.^[^ ^14^ ^]^ This leads to a situation where sweat accumulation occurs within the microclimate when the rate of sweat evaporation is slower than that of sweat secretion. This excessive humidity results in an increase in the heat index (*HI*), which is a measure of thermal comfort taking into account air temperature and relative humidity.^[^ ^15^ ^]^ *HI* can be obtained^[^ ^16^ ^]^:

(2) $$\begin{array}{c} HI\;=\;-42.379+2.04901523T+10.14333127R-0.22475541TR\\ -6.83783\times 10^{-3}T^{2}-5.481717\times 10^{-2}R^{2}+1.22874\times 10^{-3}T^{2}R\\ +8.5282*10^{-4}TR^{2}-1.99*10^{-6}T^{2}R^{2} \end{array}$$

Where *T* is ambient temperature (°F), *R* is relative humidity (%). There is a positive correlation between the *HI* and the *R*. Therefore, efficient moisture management in textiles plays a crucial role in evaporating sweat from the skin and keeping it dry and comfortable.

As a protective barrier, garments play a crucial role in personal thermal‐moisture management. Undoubtedly, integrating cooling technologies into clothing presents a promising approach for practical applications. Personal cooling textiles can provide localized cooling to specific body regions with high accuracy, which can be tailored to meet unique thermal comfort needs according to various environments. Personal cooling textiles can be broadly categorized as either “active” or “passive”.^[^ ^17^ ^]^ Active cooling textiles incorporate cooling systems directly into the garments, such as ventilation/air conditioning platforms, liquid coolants, and thermoelectric (TE) cooling systems. They regulate temperature by driving the heat transfer through the input of electrical or mechanical energy.^[^ ^18^ ^]^ However, active cooling textiles often come with drawbacks of higher costs, energy‐inefficient, and discomfort to wear. Passive cooling textiles rely on natural physical processes without the need for external energy input, which is more suitable for daily use with the advantages of low cost, energy efficiency, and a well‐fitted design. Radiative cooling textiles are designed to efficiently emit the body's infrared heat radiation and minimize heat absorption from the surroundings by optimizing composition and surface properties.^[^ ^19^ ^]^ Thermal conductive cooling textiles facilitate the transfer of heat from the body to a colder object or the surrounding environment. Radiative cooling textiles and thermal conductive cooling textiles rely heavily on ambient temperature for their cooling capabilities. The human body maintains an approximate surface temperature of 33 °C.^[^ ^20^ ^]^ These textiles dissipate heat effectively when the ambient temperature is lower than the body's surface temperature. Additionally, the efficiency of radiative cooling technology is closely related to geographical locations and climates. Specifically, radiative cooling technology is more suitable for application in a clear day and dry climate because clouds and high humidity can reduce the transparent atmospheric window, which will affect the cooling performance.^[^ ^21^, ^22^ ^]^ Conversely, in hot environments (>33 °C), the body directly absorbs heat from the surroundings. At this point, the cooling effectiveness of radiative cooling and thermal conduction cooling textiles diminishes significantly. In high temperatures, the skin primarily regulates body temperature through perspiration. Evaporative cooling textiles, characterized by unique fabric constructions, facilitate the evaporation of sweat from the skin's surface, thus enhancing cooling efficiency. In high temperatures, the skin primarily regulates body temperature through perspiration. Evaporative cooling textiles, characterized by unique fabric constructions, facilitate the evaporation of sweat from the skin's surface, thus enhancing cooling efficiency. Lower humidity and high airflow velocity are equally pivotal factors that accelerate the rate of sweat evaporation on textiles and cooling potential. Furthermore, smart textiles can respond to surrounding stimulates, providing personalized thermal management and regulating both human body radiation and humidity dynamically. These textiles have emerged as a promising solution to cope with changing circumstances.

**Figure 1:**
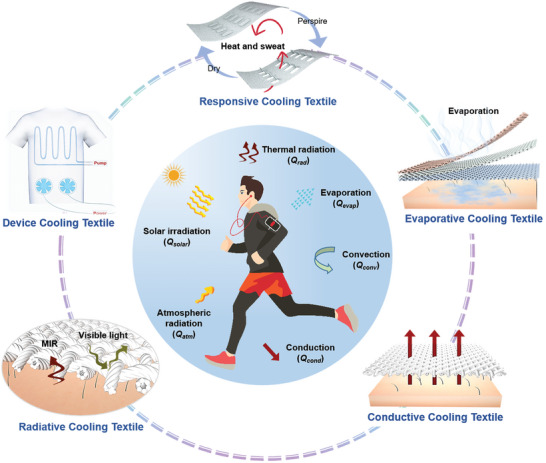
Schematic for heat transfer mechanisms of a human body in an outdoor environment and five types of cooling textiles for personal thermal management.

This review provides a comprehensive summary of cooling textiles, classifying them according to heat transfer mechanisms, including wearable devices, radiative cooling textiles, thermal conductive cooling textiles, evaporative cooling textiles, and dynamic responsive cooling textiles (as illustrated in **Figure** **1**). It emphasizes the fundamental principles and process routes of personal cooling textiles, shedding light on the latest research advancements and future directions in this field. The development and integration of advanced cooling technologies into textiles have paved the way for efficient and sustainable cooling solutions. By harnessing the power of innovative materials, design concepts, and manufacturing techniques, personal cooling textiles have the potential to revolutionize industries such as sports, outdoor activities, healthcare, and occupational safety. With their ability to provide localized and personalized cooling, these textiles can enhance comfort and well‐being while minimizing energy consumption and reducing environmental impact. Furthermore, the review analyzes the challenges and opportunities that lie ahead in terms of functionality and application, offering valuable insights for further development and innovation.

## 2 Device Cooling Textiles

In the human body, metabolic heat is generated and transported through blood circulation to the body's surface, where it is dissipated to the surrounding environment.^[^ ^2^ ^]^ With the emergence of wearable cooling systems, providing localized cooling to specific body regions with high accuracy is possible.^[^ ^23^ ^]^ These personal cooling systems can be tailored to meet each individual's unique thermal comfort needs and health conditions, which are widely used in various special work scenarios, such as sports, military, aerospace, and healthcare. This part provides an overview of the design of cooling devices, including air ventilation textiles, liquid cooling textiles, and TE cooling systems.

### 2.1 Air Ventilation Garments

**Figure 2:**
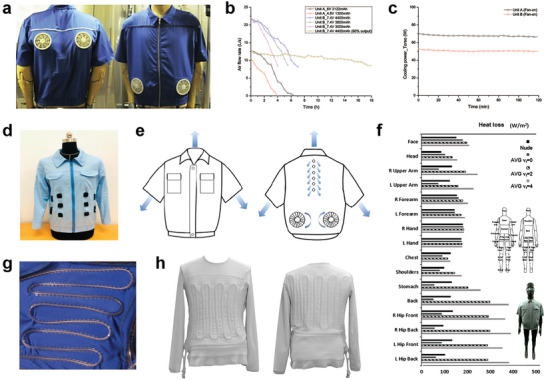
Device cooling textiles based on air ventilation and liquid cooling. a) AVG with fans located at different torso sites. Reproduced with permission.^[^ ^25^ ^]^ Copyright 2013, Elsevier. b) The airflow rate of the ventilation unit. c) Torso cooling power provided by the ventilation unit. Reproduced with permission.^[^ ^26^ ^]^ Copyright 2017, Elsevier. d) AVG with small fans. Reproduced with permission.^[^ ^27^ ^]^ Copyright 2023, MDPI. e) AVGs with two fans and openings located at the back site. (f) Heat loss values of body thermal zones dressed with AVG. Reproduced with permission.^[^ ^31^ ^]^ Copyright 2021, Elsevier. g) Tubes are integrated by Zigzag stitching in the LCG. Reproduced with permission.^[^ ^34^ ^]^ Copyright 2010, Taylor & Francis. h) LCG with a tube system distributing a cooling liquid. Reproduced with permission.^[^ ^36^ ^]^ Copyright 2017, Elsevier.

Air ventilation garments (AVGs) are designed to enhance the evaporation and convective heat transfer of sweat by facilitating airflow within the microclimate surrounding the body.^[^ ^24^ ^]^ AVGs are usually equipped with fans to promote air circulation. Zhao et al. designed a set of AVGs equipped with fan units positioned at different sites on the torso (**Figure** **2**a), offering valuable reference and guidance for industrial personal cooling systems.^[^ ^25^ ^]^ Yi et al. evaluated the properties of the portable AVGs, particularly focusing on air flow rates and cooling power. The ventilation units achieved a high airflow rate of 8–22 L s^−1^ for 7 h, along with a cooling power of 68 W (Figure 2b,c), demonstrating an outstanding cooling effect.^[^ ^26^ ^]^ While the fans with large sizes (diameter of 9.8 or 10 cm) and weight lead to discomfort for the wearer. To satisfy the need for cooling effect and comfort, Zhao et al. designed AVGs with smaller fans and explored the effect of fan quantity and placement on cooling performance (Figure 2d). AVGs equipped with small fans allowed for both thermal comfort and comfort simultaneously.^[^ ^27^ ^]^

Except for ventilation units, the cooling performance of AVGs is primarily determined by clothing features, such as clothing sizes and gas permeability.^[^ ^28^, ^29^, ^30^ ^]^ The heat exchange efficiency of typical AVGs with ventilation fans is limited due to the lack of air outlets. Ferraro et al. addressed this limitation by incorporating six ventilation holes at the upper back of the AVG (Figure 2e). Two fans located on the posterior‐lateral side of the manikin provide a combined airflow directed towards the upper torso, enabling the air to exit through the ventilation holes and improving convection heat transfer. Figure 2f illustrates the heat loss from different thermal zones of the body when using AVGs with varying fan velocities (*v*_f_). The results indicate significant heat loss in the torso area.^[^ ^31^ ^]^ AVGs offer a potential solution for mitigating heat stress in individuals working where the air temperature is lower than the skin temperature.

### 2.2 Liquid Cooling Garments

Liquid cooling garment (LCG) consists of a fabric integrated with tubes that circulate a stream of cooling liquid, allowing for the conduction of body heat to the cold liquid.^[^ ^32^ ^]^ Liquid medium with high specific heat capacities, such as water and ethylene glycol, is chilled and pumped through the garment using an external hardware system carried in a backpack.^[^ ^33^ ^]^ LCGs were originally developed for astronauts to reduce heat stress in extreme environments, for example, an extravehicular activity space suit with LCG was used on the Apollo flights in 1969.^[^ ^12^ ^]^ With the evolution of liquid cooling technology, LCGs have been widely used in various fields, such as fire protection, construction, and even in people's daily lives.

The original design of full‐body LCGs involved stitching tubing onto the fabric (Figure 2g), tubes contact the skin directly, leading to potential overcooling in specific body areas and constriction of blood vessels.^[^ ^34^ ^]^ Subsequent studies have focused on improving the design and functionality of LCGs.^[^ ^35^ ^]^ For example, Bartkowiak et al. developed an active liquid cooling garment with a tube system placed within vertical channels between elastic layers of a knitted spacer module fabric (Figure 2h). This design eliminated the issue of local overcooling and the close fit of the coolant distributing system to the body enhanced heat conduction and cooling efficiency.^[^ ^36^ ^]^ Guo et al. took a comprehensive approach, combining physiological, biomedical, engineering, and ergonomic principles to develop a detailed theoretical model for heat transfer from human skin to the environment through LCGs. This model integrated all relevant factors and allowed for the quantitative assessment of LCG performance.^[^ ^37^ ^]^

### 2.3 Thermoelectric Cooling Systems

**Figure 3:**
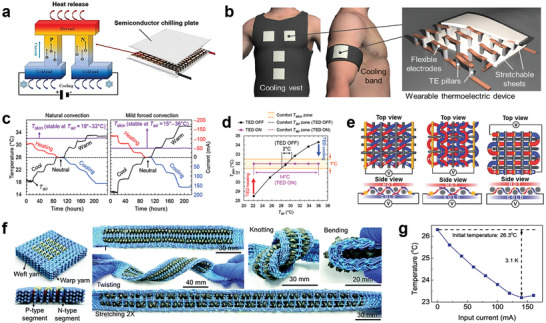
Device cooling textiles based on thermoelectric cooling. a) Schematic diagram of TE cooling system. Reproduced with permission.^[^ ^38^ ^]^ Copyright 2022, Elsevier. b) Schematic illustration of cooling garments with TE devices. c) Thermoregulation measurement results with natural convection and mild forced convection (5 km h^−1^ wind), respectively. d) The TE system broadened the comfortable ambient temperature zone from 2 to 14 °C. Reproduced with permission.^[^ ^45^ ^]^ Copyright 2019, The American Association for the Advancement of Science. e) Schematic illustrations of realized zigzag‐stitch, garter‐stitch, and plain‐weave TE textiles, respectively. Reproduced with permission.^[^ ^46^ ^]^ Copyright 2016, John Wiley & Sons. f) Diagrams of the TE textile. g) The temperature change of the TE with different applied currents when the initial temperature is 26.3 °C. Reproduced with permission.^[^ ^47^ ^]^ Copyright 2022, Royal Society of Chemistry.

TE cooling system is a solid‐state energy converter that utilizes thermocouples connected electrically in series and thermally in parallel (**Figure** **3**a).^[^ ^38^ ^]^ It operates based on the Peltier–Seebeck effect, where electric energy is converted into refrigeration.^[^ ^39^, ^40^ ^]^ The underlying principle of TE cooling involves the generation of induced thermal diffusion fluxes when charge carriers flow through a structure composed of two conducting media. These thermal diffusion fluxes result in temperature heterogeneity within the structure, generating a cooling or heating effect.^[^ ^41^ ^]^ TE cooling systems offer several advantages over conventional cooling devices, including their small size, lightweight, absence of working fluids, operation with direct current, and high cooling efficiency.

In recent years, there has been significant interest in wearable TE cooling systems for personal thermal management.^[^ ^42^, ^43^, ^44^ ^]^ Hong et al. designed a flexible and wearable TE cooling system by incorporating rigid inorganic TE pillars between stretchable elastomer sheets (Figure 3b). This design achieved a substantial cooling effect of over 10 °C on the skin without the need for external heat sinks (Figure 3c). The device demonstrated cooling capabilities for more than 8 hours and expanded the comfortable ambient temperature range from 2 to 14 °C (Figure 3d).^[^ ^45^ ^]^ Another innovative approach was taken by Lee et al., who fabricated a flexible and lightweight TE yarn using electrospun polymer nanofiber cores coated with n‐type/p‐type semiconductor sheaths interconnected by gold interconnects. These TE yarns could be knitted and woven in series or parallel with various structures such as zigzag, garter‐stitch, or plain (Figure 3e). The plain‐structured TE textile exhibited a significantly higher output power of 0.62 W m^−2^ at a temperature difference of 55 °C.^[^ ^46^ ^]^ Zheng et al. employed a weaving machine to design and produce woven Bi_2_Te_3_‐based TE strings for the fabrication of a TE textile (Figure 3f). The TE textile demonstrated a noticeable cooling effect due to the high Seebeck coefficient, electrical conductivity, and low thermal conductivity of the TE materials. As shown in Figure 3g, it continuously and reliably generated solid‐state cooling of 3.1 K in a quiescent air environment.^[^ ^47^ ^]^

Device cooling textiles offer significant advantages in terms of high cooling efficiency and have found widespread use across various industries. However, these textiles often suffer from major drawbacks including bulkiness, rigidity, and inconvenience during washing, which restrict the wearer's freedom of movement. Besides, active cooling systems are heavily dependent on electrical power, leading to high costs and low energy efficiency. These factors have posed significant challenges to the commercialization and widespread adoption of active cooling textiles in the market. In contrast, passive cooling presents an alternative strategy that eliminates the need for complex piping systems, equipment, and external energy sources.^[^ ^48^ ^]^ In the following sections, we will primarily focus on four types of passive cooling textiles. The aim is to provide a comprehensive overview of the materials used and the structural designs employed in passive cooling textiles, inspiring further research and development in this field and exploring their potential applications.

## 3 Radiative Cooling Textiles

**Figure 4:**
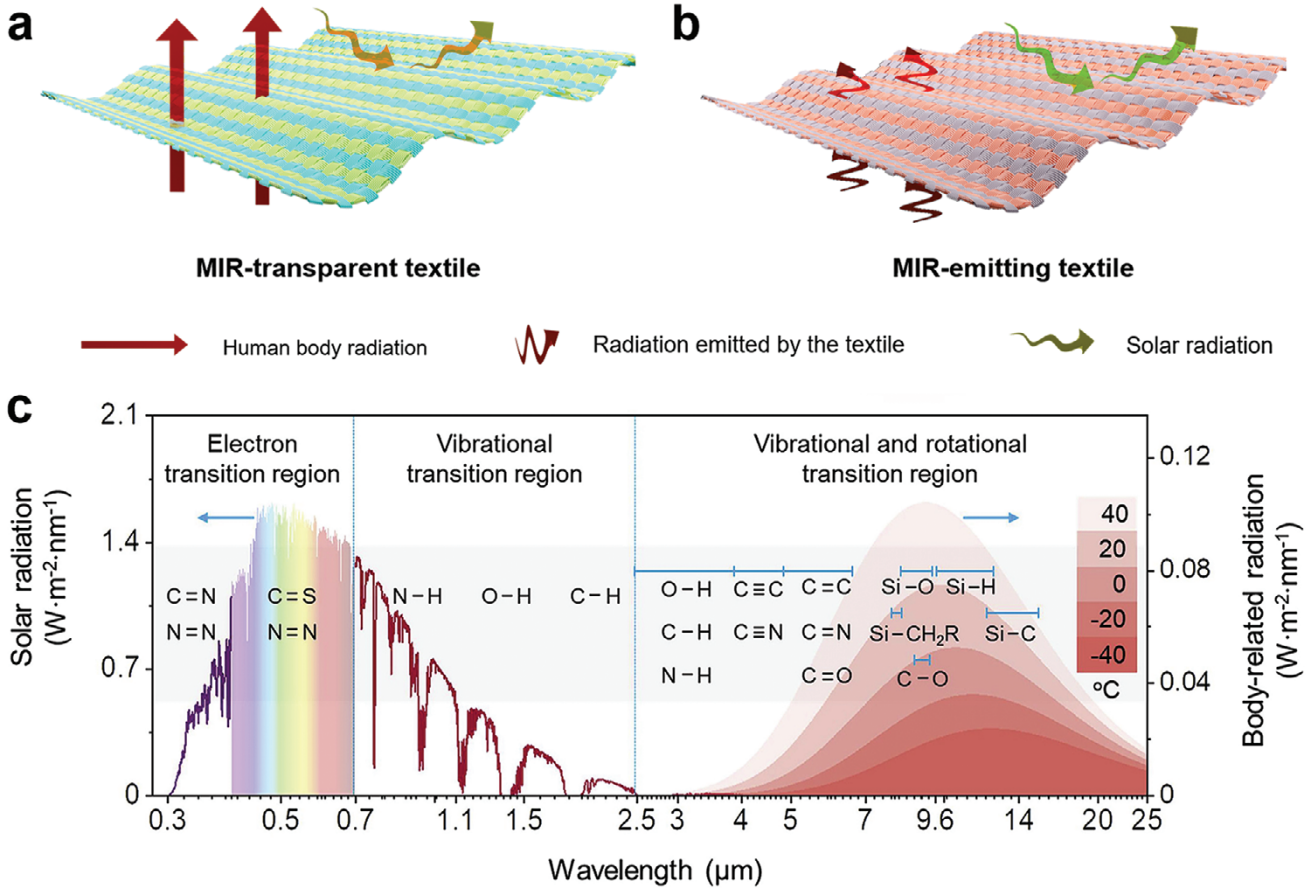
a) MIR transparent radiative cooling textile. b) MIR emissive radiative cooling textile. c) Spectra of solar‐ and body‐related radiation and spectral in typical polymers. Reproduced with permission.^[^ ^55^ ^]^ Copyright 2021, Elsevier.

Objects can transfer heat to outer space through the “atmospheric window” (8–13 µm) in the form of electromagnetic radiation.^[^ ^49^ ^]^ This phenomenon, known as radiative cooling, is a highly energy‐efficient and sustainable cooling technology that takes advantage of the earth's natural cooling process.^[^ ^50^, ^51^ ^]^ The human body is a nearly perfect “black body” with an emissivity of 0.98. It emits thermal radiation in the mid‐infrared (MIR) wavelength range of 7–14 µm, which coincides with the “atmospheric window”.^[^ ^52^, ^53^ ^]^ It is worth noting that at an ambient temperature of 25 °C, over 60% of the total heat loss of the human body occurs through radiation.^[^ ^54^ ^]^ Radiative cooling textiles play a critical role in human body's radiative dissipation by regulating optical properties. Two major strategies have been proposed in the development of radiative cooling fabrics (**Figure** **4**a,b): 1) Manufacturing radiative cooling fabrics with high transmittance in the MIR band; 2) Manufacturing radiative cooling fabrics with high emissivity in the MIR band.

### 3.1 MIR Transparent Radiative Cooling Textiles

**Figure 5:**
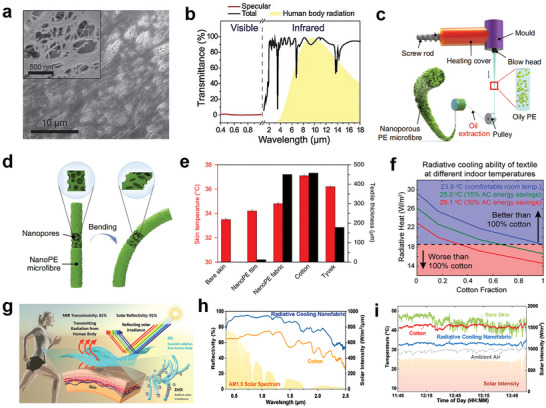
MIR transparent radiative cooling textiles. a) SEM images of nanoPE. The nanopores are only 50 to 1000 nm in diameter, ensuring high infrared transmittance. b) Total and specular transmittance of infrared and visible light for nanoPE, with an average pore size of 400 nm. Reproduced with permission.^[^ ^59^ ^]^ Copyright 2016, The American Association for the Advancement of Science. c) A schematic diagram of the manufacturing process for the nanoPE microfiber. d) An illustration showing the nanoscale porosities adapting to a bent deformation. e) Artificial skin temperature measurement of nanoPE fabric and others. Reproduced with permission.^[^ ^60^ ^]^ Copyright 2018, Springer Nature. f) Outward total radiative heat flux versus cotton fraction for different ambient temperatures. Reproduced with permission.^[^ ^61^ ^]^ Copyright 2016, American Chemical Society. g) Schematic of the radiative cooling nanofabric embedded with ZnO nanoparticles. h) UV–vis–NIR reflectance spectra of the radiative cooling nanofabric and cotton. i) Simulated skin temperatures with the radiative cooling nanofabric, cotton, and bare skin measured using thermocouples on a clear summer day. Reproduced with permission.^[^ ^64^ ^]^ Copyright 2022, American Chemical Society.

MIR transparent radiative cooling textiles can minimize the body heat radiation absorption from textiles and maximize the heat dissipation rate from the body towards the cold outer space (Figure 4a). However, most of the infrared absorption wavelength of common textile materials corresponds with the atmospheric window radiation spectrum (Figure 4c), absorbing a significant amount of infrared radiation in the spectral region of human body radiation.^[^ ^55^ ^]^ Hence, the crucial assignment for developing radiative cooling textiles is to explore materials that are transparent in the MIR wavelength range of 7–14 µm. Polyethylene (PE), composed of only aliphatic C─C and C─H bonds, behaves with high long‐wave infrared transmittance on account of the absence of polar molecular groups.^[^ ^56^, ^57^ ^]^ Therefore, PE often be used to fabricate MIR transparent fabric. For the demand of daily wearing, infrared‐transparent visible‐opaque fabric was proposed.^[^ ^58^ ^]^ Hsu et al. utilized nanoporous PE (nanoPE) film as an excellent infrared‐transparent textile for human body cooling. As shown in **Figure** **5**a,b, the interconnected pores in nanoPE correspond to the wavelength of visible light, allowing for the effective scattering of visible light while preserving its MIR transparency.^[^ ^59^ ^]^

However, PE film is by nature unsuitable for normal clothing due to the lack of wearable comfort. Subsequently, Peng et al. produced continuous nanoPE microfibers through large‐scale extrusion (Figure 5c). The nanoPE microfibers possess high air permeability, water‐wicking property, and good softness on account of nanoscale pores in the microfiber, which can be extensively adopted for clothing (Figure 5d). As shown in Figure 5e, the nanoPE fabric can lower the human skin temperature by 2.3 °C compared to cotton fabric.^[^ ^60^ ^]^ Furthermore, Catrysse et al. proposed a design for blending infrared‐opaque fibers (one‐third cotton) and infrared transparent fibers (two‐thirds polyamide (PA)) to improve the comfort of the textile, as well as preserve the opacity of the textiles at visible wavelengths.^[^ ^61^ ^]^ As shown in Figure 5f, the outward total radiative heat fluxes versus cotton fractions for different ambient temperatures were investigated. This confirms that the design of photonic structure textiles can offer suitable localized thermal management, which provides a guideline for the manufacture of MIR transparent radiative cooling textiles with wearable comfort.

When exposed to direct sunlight, it confronts with the challenge of experiencing significant heating from solar irradiance.^[^ ^52^ ^]^ Therefore radiative cooling textiles with the capacity of reflecting sunlight were designed to avoid solar heating.^[^ ^62^ ^]^ High refractive materials (e.g. zinc oxide (ZnO)), with a particular size range, can have a strong resonant light scattering effect in the visible spectral range based on Mie theory.^[^ ^63^ ^]^ Cai et al. prepared porous ZnO‐PE fibers via phase separation melt spinning. ZnO has not only a high refractive index (n≈2), but also little absorption from visible to MIR wavelengths. The ZnO‐PE fibers show high reflectivity of more than 90% in the solar light region and high transmissivity of 80% in the human body thermal radiation.^[^ ^52^ ^]^ Iqbal et al. manufactured a radiative cooling nanofabric by incorporating ZnO into PE through electrospinning technology (Figure 5g). The nanofabric can reflect 91% of solar irradiance while transmitting infrared, which cools the simulated skin by 9 °C compared to cotton fabrics (Figure 5h,i).^[^ ^64^ ^]^

Few polymer materials with high infrared transmittance except for PE have been reported to prepare textiles. Novel infrared‐transparent polymers should be invented to accommodate the various needs for wearability, such as flexibility, moisture transport, and air permeability. A general guideline is to search for PE derivative materials with a high content of crystalline aliphatic segments.^[^ ^65^ ^]^

### 3.2 MIR Emissive Radiative Cooling Textiles

In addition to MIR transparent textiles possessing the radiation cooling effect, controlling the surface emissivity of textiles is also an effective approach for temperature regulation.^[^ ^66^ ^]^ As illustrated in Figure 4c, emission in the MIR region is typically associated with molecular bonds vibrating of polymers at 8–13 µm, such as C─O─C (1260–1110 cm^−1^), C─OH (1239–1030 cm^−1^), ─CF_3_ (1148 cm^−1^) and Si─O─Si (1100 cm^−1^).^[^ ^67^, ^68^ ^]^ Traditional fabrics are known to exhibit high absorptivity in the MIR band attributed to the properties of their constituent materials. According to Stefan‐Boltzmann's law, traditional textiles emit infrared radiation with significantly lower power compared to solar irradiation.^[^ ^65^ ^]^ Therefore, textiles can achieve cooling effects through the selective prevention of heat absorption in the wavelengths beyond the atmospheric window.^[^ ^52^ ^]^ Due to the high emissivity, the MIR emissive radiative cooling textiles can reflect the NIR radiation emitted by incoming solar rays, while simultaneously enhancing the emission of thermal radiation to cold outer space in the MIR range through the transparent atmospheric window. This process ensures that the overall heat gain from the surrounding environment remains lower than the total heat loss facilitated by the strong reflectance and emissivity of the textile.

**Figure 6:**
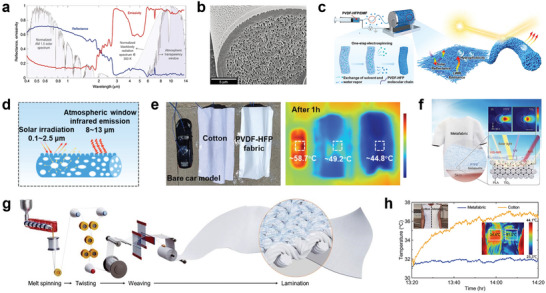
MIR emissive radiative cooling textiles. a) Integrated hemispherical reflectance and emissivity spectra of a comet moth cocoon fiber from the visible to the MIR. b) SEM images of a regenerated silk fiber cross‐section containing a high density of voids. Reproduced with permission.^[^ ^75^ ^]^ Copyright 2018, Springer Nature. c) Schematic of facile one‐step electrospinning process for preparing PVDF‐HFP fabric consisting of nanosphere structures and hierarchically porous fibers. d) Schematic illustration of the working principle of designing the rough convex nanosphere and porous structure scattering the sunlight to enhance reflectance and the intrinsic characteristics radiating heat in the long‐wave infrared. e) Digital pictures and thermal images of three identical car models at different states, respectively. Reproduced with permission.^[^ ^76^ ^]^ Copyright 2023, Elsevier. f) Schematic of a metafabric for radiative cooling. g) The fabrication process of the metafabric. h) Temperature tracking for skin under different fabrics in direct sunlight. The insets show photographs and thermal images of the volunteer wearing a homemade vest. Reproduced with permission.^[^ ^78^ ^]^ Copyright 2021, The American Association for the Advancement of Science.

Drawing inspiration from biological microstructures in nature, the construction of textile‐based photonic radiative cooling structures for thermoregulation has emerged as a promising strategy for passive radiative cooling.^[^ ^69^, ^70^, ^71^, ^72^ ^]^ Optimizing the nano‐ or micro‐structures to block energy input from solar radiation using the Mie effect is a common strategy.^[^ ^73^ ^]^ Silkworm silk fibers possess passive cooling properties attributed to the strong reflectivity generated by Anderson's localization of biomolecules.^[^ ^74^ ^]^ These fibers contain a high density of randomly distributed air voids with sizes comparable to visible and NIR wavelengths, enhancing the solar reflectance of fibers (**Figure** **6**a). To explore alternative materials of natural silk, Shi et al. investigated biomimetic regenerated silk fibroin and polyvinylidene fluoride (PVDF) fibers embedded with a high density of voids (Figure 6b). These fibers demonstrated the ability to guide light along the longitudinal direction through Anderson localization, exhibiting favorable radiative cooling characteristics.^[^ ^75^ ^]^ Cheng et al. developed a nanosphere‐structured hierarchically porous fibrous fabric made of PVDF‐hexafluoropropylene (HFP) through an electrospinning process based on water vapor‐induced phase separation (Figure 6c). The PVDF‐HFP fibers contain C─F groups that exhibit strong infrared emission within the atmospheric transparent window. Additionally, the porous fibrous structure exhibits a remarkably high solar reflectance of 93.7% and an extremely high UV reflectance of over 97% (Figure 6d). As shown in Figure 6e, the remarkably practical daytime radiative cooling performance was confirmed by a temperature drop of ≈13.2 °C compared with the one covered with cotton under sunlight.^[^ ^76^ ^]^

Furthermore, as scatterers with high refractive indices, inorganic compounds (e.g., SiO_2_, titanium dioxide (TiO_2_)) can leverage the collective effect of multiple Mie resonances to minimize the mean free path of solar scattering.^[^ ^9^, ^77^ ^]^ Zeng et al. prepared composite microfibers consisting of TiO_2_ by melt spinning technology and wove them into a multi‐layer metafabric (Figure 6f,g). The metafabric provides high reflectivity of 92.4% across the entire visible‐to‐NIR band, which could cool 4.8 °C lower than the commercial cotton fabric (Figure 6h).^[^ ^78^ ^]^

Radiative cooling textiles can reduce the energy demand for cooling. However, there is currently a lack of comprehensive analysis of the energy‐saving benefits and environmental impacts of integrating passive radiative cooling textiles into sportswear and other apparel products. This area deserves further exploration to facilitate the integration of this emerging technology into everyday lifestyles.

## 4 Thermal Conductive Cooling Textiles

Heat will spontaneously transfer from a hot body to a cold body according to the second law of thermodynamics. Thermal conductive cooling is also an efficient heat dissipation method for fabrics. When the environmental temperature is lower than the body surface temperature, the heat flow caused by the temperature gradient will flow from the inner side of the fabric to the outer surface at a macro level. At a micro level, thermal conduction occurs when internal energy is transferred by the movement of electrons or lattice vibrations within a material, and through microscopic collisions of particles between two adjacent materials from a high‐temperature region to a low‐temperature region.^[^ ^79^ ^]^ Thermal conductivity is a parameter that characterizes the ability of a material to conduct heat, which is dependent on material properties, morphological structures, and aggregation structures.^[^ ^80^, ^81^ ^]^ There are two different approaches to enhance thermal conduction mechanisms: 1) Manufacturing fibers with high crystallinity and orientation; 2) Incorporating fillers of high thermal conductivity with textiles.

### 4.1 Textiles with High Crystallinity and Orientation

**Figure 7:**
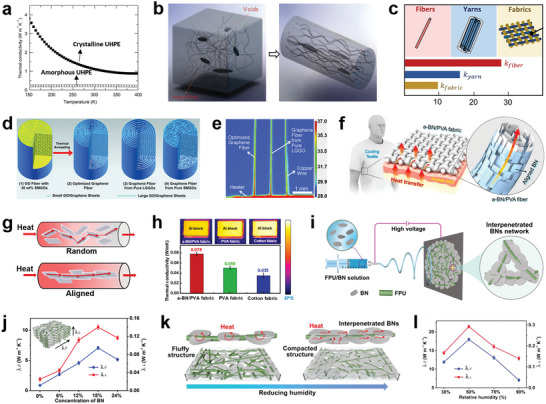
Thermal conductive cooling textiles with high crystallinity, orientation, and enhanced thermal conductivity. a) Thermal conductivity of crystalline and amorphous UHPE at 0.05 GPa from 150 to 400 K. Reproduced with permission.^[^ ^85^ ^]^ Copyright 2014, Elsevier. b) Bulk PE containing chain ends, entanglements, voids and defects, and stretched PE microfiber. Reproduced with permission.^[^ ^90^ ^]^ Copyright 2010, Springer Nature. c) Thermal conductivity research of the UHMW‐PE fiber, yarns, and woven fabrics, respectively. Reproduced with permission.^[^ ^93^ ^]^ Copyright 2020, American Chemical Society. d) Schematics of the “intercalated” structure of the GO fibers and graphene fibers. e) A thermal image showing rapid heat transport on the optimized graphene fiber. Reproduced with permission.^[^ ^100^ ^]^ Copyright 2015, The American Association for the Advancement of Science. f) Schematic illustration of the BN/PVA composite fabric. g) Schematic illustration of the proposed thermal conduction model of the a‐BN/PVA fiber. The aligned BNNSs form continuous thermally conductive pathways along the fiber direction. The red lines indicate heat transfer paths. h) Measurements of the thermal conductivities of the a‐BN/PVA fabric compared with others. Reproduced with permission.^[^ ^105^ ^]^ Copyright 2017, American Chemical Society. i) Schematic illustration of the fabrication and structure of thermos‐conductive nanofibrous membranes. j) In‐plane and cross‐plane thermal conductivity of the FPU/BN membrane. k) Diagram of heat conduction of the FPU/BN membranes fabricated under high and low humidity. l) In‐plane and cross‐plane thermal conductivity of membranes fabricated under different relative humidities. Reproduced with permission.^[^ ^106^ ^]^ Copyright 2020, American Chemical Society.

Thermal conduction of amorphous polymers primarily relies on phonon transmission, which is the transfer of energy generated by lattice structure vibration. For crystalline polymers, electron motion and lattice vibration or their combination are the primary modes of thermal conduction.^[^ ^82^ ^]^ Generally, crystalline polymers exhibit higher thermal conductivity than amorphous polymers due to the ordered structure. Traditional fibers are an aggregation of crystalline and amorphous regions. The amorphous arrangement of the molecular chains reduces the mean free path of heat‐conducting phonons, resulting in low thermal conductivity coefficients for traditional textiles.^[^ ^83^ ^]^ Cotton fiber, for example, has a thermal conductivity coefficient of 0.026–0.065 W m^−1^ K^−1^.^[^ ^84^ ^]^ Hence, the thermal conductivity of organic polymer fibers is highly dependent on the crystallinity. Yu et al. investigated the thermal conductivity of low‐density PE, high‐density PE, and ultra‐high‐density PE (UHPE), respectively (**Figure** **7**a), and observed that increased crystallinity reduces thermal resistance.^[^ ^85^ ^]^ Although increasing crystallinity is a common way to improve the thermal conductivity of polymers, the room for improvement is very limited due to the already high crystallinity of fibers.

As heat transports more easily along polymer chains than perpendicular to the polymer chain, polymers exhibit significant anisotropy in their thermally conductive properties. It is expected that aligning the orientated crystal structure of polymers by drawing can increase its thermal conductivity significantly.^[^ ^86^, ^87^, ^88^, ^89^ ^]^ Shen et al. fabricated ultra‐drawn PE nanofibers by stretching to restructure the polymer chains, improving the fiber quality toward an ideal single crystalline fiber (Figure 7b). These nanofibers exhibited a high thermal conductivity of 104 W m^−1^ K^−1^.^[^ ^90^ ^]^ Chien et al. prepared semicrystalline PA nanofibers, and the configurational order of the polymer was improved by annealing. The fibers behaved with a high thermal conductivity of 59.1 W m^−1^ K^−1^, which could improve the thermal comfort of the human body.^[^ ^91^ ^]^ Zhu et al. handled an ultrahigh‐molecular‐weight polyethylene (UHMW‐PE) microfiber by heat‐stretching. The thermal conductivity of the fiber enhanced from 21 to 51 W m^−1^ K^−1^. The enhancement of thermal conductivity can be attributed to the reconstruction of the amorphous region. Heat stretching enhanced the amorphous alignment.^[^ ^92^ ^]^ Candadai et al. reported the thermal characterization of fabrics constructed from UHMW‐PE fibers for the first time.^[^ ^93^ ^]^ UHMW‐PE fabrics show a very high thermal conductivity, which only reduced by a factor of 3 as microfibers woven into 2D fabrics (Figure 7c).

### 4.2 Textiles Integrated with High Thermal Conductive Fillers

The thermal conductivity of fibers is affected by both their morphology and material properties. Natural fibers, such as cotton and wool fibers, have low thermal conductivity due to their unique structures. Mature cotton fibers have hollow‐channeled lumens, and wool cashmere fibers have a hollow medulla structure and a large curl curvature, which create pockets of still air. As is well‐known, air is a poor conductor of heat, which acts as a barrier to body heat dissipation. To improve the thermal conductivity of textiles, fillers with highly thermal conductive can be introduced to establish the continuous thermal conduction channel, encouraging heat loss from the body.^[^ ^94^ ^]^ Inorganic high‐conduction materials like graphene, carbon nanotubes (CNT), boron nitride (BN), nanocrystalline metal, and metal oxides, etc., are used for a variety of high‐temperature heat transfer and heat dissipation due to their high thermal conductivity and stability.^[^ ^95^, ^96^, ^97^ ^]^ Recent breakthroughs in thermal conductive cooling textile research have been achieved through the development of appropriate composite materials with high thermal conductivity.

Graphene, a monolayer of sp^2^‐hybridized carbon atoms arrayed in a honeycomb lattice, exhibits an extraordinary thermal conductivity (2000–5000 W m^−1^ K^−1^), which can generate thermal transmission for passive cooling.^[^ ^95^, ^98^, ^99^ ^]^ Xin et al. mixed large‐sized GO (LGGO) (average size of 23 µm) and small‐sized GO (SMGO) (average size of 0.8 µm) to prepare graphene fibers via a wet‐spinning strategy.^[^ ^100^ ^]^ LGGO sheets form a highly aligned backbone, whereas SMGO sheets fill the space and voids between LGGO sheets (Figure 7d). The directional alignment of graphene sheets and the compactness of the fiber structure determined the rapid heat transport of the graphene fiber (Figure 7e).

BN has been traditionally considered an effective material in thermal management applications due to its high thermal conductivity (2000 W m^−1^ K^−1^).^[^ ^101^, ^102^ ^]^ BN with high aspect‐ratio structures exhibits anisotropic thermal conductivity, whose thermal conductivity in a longitudinal direction is usually much higher than its transverses.^[^ ^103^ ^]^ The design of BN nanosheets (BNNSs) highly aligned is a good strategy for achieving high thermal conductivity of fabrics.^[^ ^104^ ^]^ Gao et al. fabricated the highly aligned BN/poly(vinyl alcohol) (PVA) composite fibers via a 3D printing method (Figure 7f). The highly aligned and interconnected BNNS established an effective heat transfer pathway (Figure 7g). The fabric improves the thermal conductivity of the composite fabric by 55% over commercial cotton fabric (Figure 7h), resulting in an attractive cooling effect.^[^ ^105^ ^]^ Yu et al. construct highly thermos‐conductive nanofibrous membranes via one‐step electrospinning, which causes BN to be linked with each other along fluorinated polyurethane (FPU) nanofibers (Figure 7i). The resultant FPU/BN nanofibrous membranes demonstrated an outstanding thermal conductive cooling performance with an ultrahigh in‐plane thermal conductivity of 17.9 W m^−1^ K^−1^ and cross‐plane thermal conductivity of 0.29 W m^−1^ K^−1^ (Figure 7j). It improved that the relative humidity of fabrication would affect the thermal conductivity. The mechanism is presented in Figure 7k,l. The reduced relative humidity brought about a compacted structure, resulting in interpenetrated BN networks, which engender large contact areas through in‐plane/cross‐plane direction to transfer heat readily.^[^ ^106^ ^]^

To summarize, the thermal conductivity of textiles is mainly influenced by their compositions and structures. Thermal conductivity is an anisotropic characteristic, meaning that factors such as aspect ratio, dimensions, concentration of fillers, and the arrangement of nanofillers in polymers would directly affect the thermal performance of composites. Here, the discussion is mainly focused on fibers and yarns, because the thermal conduction mechanism becomes more complex in fabrics with 3D structures compared to that in 1D fibers/yarns. Apart from the parameters of fibers and yarns, the fabric's structure, thickness, porosity, and density also contribute to determining the fabric's thermal conductivity.

Additionally, the thermal conduction ability of textiles is highly sensitive to humidity and moisture content. The expansion of fabric due to moisture absorption, which results in alterations in material and structural properties, has a notable impact on thermal conductivity. Wetting considerably enhanced the heat transfer coefficient of the fabrics. The effective thermal conductivity of completely wet cotton fibers ranges between 0.65 and 0.70 W m^−1^ K^−1^, a range comparable with that of liquid water itself.^[^ ^107^ ^]^ However, the accumulation of moisture and sweat will lead to a decrease in air permeability and a feeling of being sticky, resulting in the reduction of thermal‐wet comfort.^[^ ^108^ ^]^ Therefore, thermal conductive cooling behaves inconspicuous cooling effect in a high temperature and humidity environment.

## 5 Evaporative Cooling Textiles

The mechanism of evaporative cooling textiles involves the absorption of moisture from the microclimate through the fabric, maintaining dryness on the skin. Simultaneously, the sweat is transferred to the outer surface of the fabric, facilitating its evaporation. Evaporative cooling, as a thermodynamic process, is a highly effective and sustainable method for air cooling.^[^ ^109^ ^]^ As sweat evaporates from the skin, it absorbs energy and converts sensible heat to vapor enthalpy, resulting in a drop in body temperature. Upon evaporative cooling based on pure water, a cooling power density of 320 W m^−2^ can be achieved, which is much higher than the theoretical limit of radiative cooling (150 W m^−2^).^[^ ^10^ ^]^ An adult can produce up to 1.5 L h^−1^ of sweat in a hot environment. At this time, evaporative heat dissipation accounts for more than 75% of the total heat. Even in a mild state, ≈20% of heat loss in a dry human body depends on the water vapor loss of insensible perspiration.^[^ ^52^ ^]^ With the huge latent heat of water vaporization (2260 kJ kg^−1^), sweat evaporation plays an essential role in the human body's thermoregulation.^[^ ^110^ ^]^

### 5.1 Moisture Sorption‐Desorption‐Based Evaporative Cooling Textiles

Traditional textile materials usually behave low moisture absorption rates due to the lack of hydrophilic groups and high crystallinity. This limits the ability to effectively absorb moisture, resulting in sweat accumulation in the microclimate. The high saturated vapor pressure in the microclimate prevents the effective evaporation of sweat from the skin. As a consequence, this leads to increased thermal stress and heat index, ultimately reducing human comfort. Therefore, enhancing moisture absorption is a crucial technique in the development of cooling textiles.

**Figure 8:**
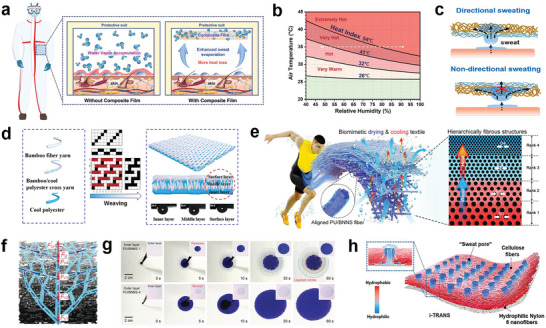
Evaporative cooling textiles with rapid moisture absorption and sweat releasing. a) Schematic illustration of the enhanced sweat evaporation by the composite film. b) Heat index chart. Reproduced with permission.^[^ ^118^ ^]^ Copyright 2022, John Wiley & Sons. (c) Mechanism diagrams of directional water transport textile. Reproduced with permission.^[^ ^121^ ^]^ Copyright 2023, Elsevier. d) Schematic diagram of the weaving technique of the three‐layer composite fabric. Reproduced with permission.^[^ ^128^ ^]^ Copyright 2022, John Wiley & Sons. e) Schematic illustrating the sweat release and heat dissipation of the biomimetic multilayer fibrous membrane as a functional textile for personal drying and cooling; the right inset is the cross‐sectional view of the hierarchically fibrous structure. f) Illustration of the spontaneous vertical wicking in the biomimetic multilayer fibrous membrane. g) Directional water transport process from the top view of the multilayers when the water droplets were dropped on the inner layer and the outer layer; insets are the bottom view. Reproduced with permission.^[^ ^129^ ^]^ Copyright 2021, John Wiley & Sons. h) Schematic of artificial sweating skin illustrating its structure and surface property. Reproduced with permission.^[^ ^132^ ^]^ Copyright 2022, John Wiley & Sons.

High hygroscopic polymers, such as polyacrylate and polyacrylamide, have been widely used as matrix materials to increase the moisture absorption of fibers.^[^ ^111^ ^]^ Li et al. developed an evaporative cooling fabric composed of a set of warp yarns and two sets of weft yarns. Water‐repellent hollow polyester yarns were utilized as the inner weft to keep the side close to the skin dry, and superabsorbent yarns were chosen as the warp and face weft to enhance the cooling duration. The fabric shows a water absorbency of 370% and an efficient cooling effect of 2–6 °C.^[^ ^112^ ^]^ Additionally, sorbents with a high moisture rate of up to 30–200% are usually used for the construction of hygroscopic textiles. Hygroscopic salts (e.g., calcium chloride (CaCl_2_), lithium bromide, and lithium chloride) have a high adsorption ability for atmospheric water.^[^ ^113^, ^114^, ^115^, ^116^ ^]^ Sun et al. demonstrated that by loading hygroscopic CaCl_2_ on the fabric, water vapor can be captured at night and evaporative cooling can be achieved during the daytime. This approach reduced the temperature of the space under the cooling fabric by 10.8 °C compared to the ambient temperature under direct sunlight.**^[^** ^117^ **^]^** However, the adsorbed atmospheric water ultimately dissolves the salts and forms an aqueous solution, which creates challenges in engineering design for atmospheric water sorbents. Yang et al. developed a super‐hygroscopic zinc‐ethanolamine complex (Zn‐complex) by adjusting the ratio of zinc ion and ethanolamine ligand.^[^ ^118^ ^]^ They then embedded the Zn‐complex in a PVA matrix to fabricate a Zn‐PVA composite film with an adsorption capacity of up to 403.7 g m^−2^. This film can be directly attached to the inside of a protective suit to absorb sweat quickly (**Figure** **8**a). As a result, the relative humidity decreases from 91.0 to 48.2%, creating a steep water vapor pressure gradient that facilitates evaporative cooling and reduces the heat index from 64.6 to 40.0 °C (Figure 8b).

### 5.2 Evaporative Cooling Textiles with Directional Water Transport

In highly active scenarios, such as exercise or physical labor, individuals perspire profusely in order to dissipate heat.^[^ ^119^ ^]^ Once the fabric reaches its absorption limit, the humidity of the microclimate will increase. Accordingly, in addition to decent hygroscopic properties, an optimal textile should also possess high wicking and evaporation abilities. The wicking ability refers to the movement of moisture within a fabric by capillary action, usually along the filament surface, diverting moisture rapidly and enlarging the wetted area. The evaporation ability directly affects the evaporative cooling efficiency. A higher wicking ability and evaporation rate can prevent the textile from becoming saturated and avoid excessive perspiration, which helps to achieve a sufficient cooling effect by using sweat in a highly efficient manner.^[^ ^120^ ^]^

In recent years, researchers have developed Janus fabrics with directional water transport features by designing the surface energy gradient structure along the thickness of the fabric. The schematic diagrams of antigravity directional perspiration through the textile are shown in Figure 8c. When the hydrophobic layer is gently touched upward, The Laplacian pressure generated by the bending liquid surface of the droplet promotes one‐way transport of the liquid in the textile. The sweat droplet effectively overcomes its gravity and passes through the hydrophobic layer to reach the hydrophilic layer and spread on it. In contrast, sweat that contacts and spreads on the hydrophilic layer is blocked by the hydrophobic layer because the textile shows a lower breakthrough pressure generated by the wetting gradient in the hydrophobic to hydrophilic direction.^[^ ^121^ ^]^ Various water management techniques have been proposed for cooling purposes, including the multiple‐layer design with a wettability gradient, the hierarchical design of multiscale interconnected pores with a capillarity gradient, and the “skin‐like” design with a conical micropore array.^[^ ^122^, ^123^, ^124^, ^125^, ^126^, ^127^ ^]^ Li et al. designed a three‐layer composite fabric with a wettability gradient for highly efficient personal drying and cooling adopting the weft‐backed weave technique (Figure 8d). The yarns in the surface layer are more hydrophilic than the yarns in the middle and inner layers, which offer good unidirectional water transmission and heat dissipation properties in the inside‐to‐outside direction.^[^ ^128^ ^]^ Inspired by the transpiration in vascular plants, Miao et al. proposed a biomimetic transpiration textile with hierarchical and interconnected network structures (Figure 8e). The schematic diagram and pictures of the water transport along the vertical direction of the multilayer fibrous membrane are shown in Figure 8f,g. The hierarchically porous structure offers capillary force which can overcome the viscous resistance and self‐gravity to transport water upward. The fabric exhibited a high water‐evaporation rate (0.36 g h^−1^), and outstanding thermal conductivities in both through‐plane (0.182 W m^−1^ K^−1^) and in‐plane (1.137 W m^−1^ K^−1^) directions, making it highly efficient for personal drying and cooling.^[^ ^129^ ^]^

Human skin is an exceptional example of a directional liquid flow material. Researchers developed a novel design strategy by mimicking the human skin to create porous gradient wettability channels across the fabric, similar to localized sweat glands.^[^ ^130^, ^131^ ^]^ In this regard, Peng et al. designed integrated cooling (i‐Cool) textiles, which integrated heat conductive pathways and water transport channels. When the human body perspires, the water transport channels efficiently wick sweat from the skin's surface and spread it onto the large‐area top surface made of fibers. The heat conductive matrix transfers human body heat efficiently to the location where evaporation takes place, thus assisting in fast evaporation. Integrated cooling (i‐Cool) shows a 2.8 °C lower temperature than cotton fabric despite the sweating rate of cotton fabric being over two–three times as much as i‐Cool. Additionally, i‐Cool exhibits better evaporative capacity and evaporative cooling efficiency compared to conventional textiles.^[^ ^120^ ^]^ They also proposed an integrated 3D hydrophilicity/hydrophobicity design for artificial sweating skin (i‐TRANS). Hydrophobic materials were employed to simulate the skin, while hydrophilic materials featuring a gradient in hydrophilicity (increasing from the bottom to the top) were used for the sweat pores. This design enables directional water transport, mimicking the natural perspiration process of the human body, where sweat moves from the bottom to the top (Figure 8h). Compared with a normal wicking layer, i‐TRANS fabric shows a 3.6 °C hypothermic effect.^[^ ^132^ ^]^

Developing strategies to further augment moisture‐wicking and water evaporation is expected to be a focus for the next generation of evaporative cooling textiles. Novel profiled fibers such as Coolmax fibers with capillary effect were developed to enhance water transport. Furthermore, the rate of water evaporation on fabrics is another critical factor influencing the efficiency of evaporative cooling textiles. Gong et al. proposed a method to enhance the moisture evaporation rate by utilizing the triboelectric field to disrupt the hydrogen‐bonding network.^[^ ^133^ ^]^ This novel approach has great potential for improving the cooling management performance of textiles. Evaporative cooling textiles have obvious advantages in hot environments and have broad application prospects in daily wear, leisure sports, and household products.

## 6 Dynamic Responsive Cooling Textiles

Smart responsive materials and structures play a crucial role in the construction of thermal‐moisture management fabrics. Unlike conventional cooling fabrics that have static structures and limited functionality, smart responsive materials possess the ability to dynamically sense and respond to environmental changes in real time.^[^ ^134^ ^]^ This dynamic responsiveness enables them to adjust their properties and behavior based on specific stimuli, allowing for the simultaneous regulation of multiple heat dissipation pathways such as conduction, convection, radiation, and sweat evaporation.^[^ ^135^ ^]^ For instance, temperature or humidity shift leads to variations in the internal structure of textiles, including alterations in pore size, thickness, and fiber structure.^[^ ^136^ ^]^ Such materials hold tremendous potential for the advancement of intelligent thermal‐moisture management systems by facilitating efficient and tailored regulation of heat and moisture transfer in response to varying environmental conditions.

Most of the thermally responsive fibers are a type of shape memory polymers, which are macromolecular structures capable of responding to heat by altering their macroscopic properties, such as color, shape, or state.^[^ ^137^ ^]^ It is worth noting that shape memory polymers typically exhibit a one‐way shape memory effect at a constant temperature, limiting their ability to adapt to continuous temperature variations. While liquid crystal elastomers and temperature memory polymers can undergo reversible deformations, they often lack the sensitivity required to detect minor temperature changes in the human body.^[^ ^138^, ^139^ ^]^ Currently, the development of dynamic responsive textiles primarily focuses on humidity‐responsive stimuli, utilizing the mechanism of anisotropic volume expansion in fibers.^[^ ^140^, ^141^ ^]^ Various moisture‐responsive materials including Nafion film, GO film, rGO film, MXene film, and poly(*N*‐isopropylacrylamide) hydrogel have been employed for this purpose. These humidity‐responsive textiles can undergo reversible shape changes in response to fluctuations in humidity levels within the microclimate, enabling effective regulation of heat and humidity. Taking inspiration from natural systems, researchers have successfully developed moisture‐ and water‐responsive actuator‐based intelligent textiles, such as pinecone effect textiles, dynamic gating textiles, and artificial muscle textiles.^[^ ^142^, ^143^, ^144^, ^145^ ^]^ The research primarily revolves around the construction of polymer films, fibers/yarns, and fabrics. Dynamic moisture‐responsive textiles aim to achieve two objectives through moisture actuation: firstly, the adaptive gating of infrared‐radiation properties, and secondly, the dynamic regulation of heat and moisture transfer within the fabric.

### 6.1 Membrane‐Based Responsive Cooling Textiles

Inspired by the pinecone structure, significant advancements have been made in the development of moisture‐responsive textiles with membrane flaps.^[^ ^146^ ^]^ One effective approach involves constructing structures with uneven expansion and contraction, which generates stress heterogeneity within the textile and leads to bending behavior when moisture is absorbed.^[^ ^134^ ^]^

**Figure 9:**
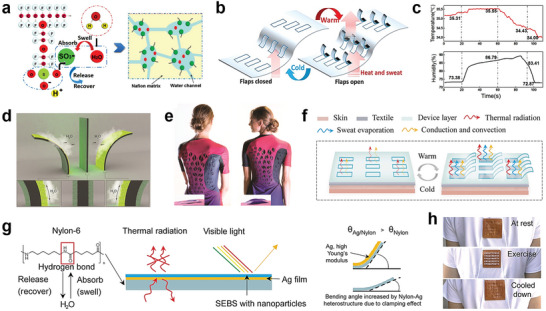
Dynamic responsive cooling textiles based on membrane structures. a) Swelling and water transport mechanism of Nafion. b) Nafion sheet schematics with openable flaps mimicking the thermo‐adaptive functionality of human skin. c) Humidity and temperature response of the “sweating” skin simulation system working at 35 °C. Reproduced with permission.^[^ ^148^ ^]^ Copyright 2017, Springer Nature. d) Shape transformation of a flat sandwich‐structured biohybrid film when exposed to moisture. e) Images of garment prototypes before exercise with flat ventilation flaps and after exercise with curved ventilation flaps. Reproduced with permission.^[^ ^149^ ^]^ Copyright 2017, The American Association for the Advancement of Science. f) Working principle of nylon/Ag heterostructure‐based multimodal wearable. g) Configuration and functional diagram of each layer of the multimodal adaptive wearable textile. h) Photos of the moisture‐responsive actuator switch before, during, and after exercise. Reproduced with permission.^[^ ^150^ ^]^ Copyright 2021, The American Association for the Advancement of Science.

Researchers have explored the fabrication of both homogeneous and heterogeneous actuators with moisture‐responsive stimuli.^[^ ^147^ ^]^ In the case of homogeneous hygroscopic textiles, a humidity gradient and volume expansion variance along the thickness direction are created by the difference in relative humidity between the two sides of the fabric, resulting in textile actuation. For instance, Zhong et al. developed a Nafion humidity‐sensitive clothing with pre‐cut flaps to mimic pores in human skin. Nafion is composed of a hydrophobic polytetrafluorethylene backbone and hydrophilic perfluoroether sulphonic acid side chains (**Figure** **9**a). As depicted in Figure 9b, one side exposed to high *RH* of the Nafion flap swells more than the other side, resulting in the flaps bending upwards and pores opening. This mechanism facilitates rapid sweat evaporation and heat release, ultimately enhancing thermal comfort for the wearer (Figure 9c).^[^ ^148^ ^]^ Similarly, Mu et al. integrated a Nafion matrix with semilunar patterns into a commercial sports shirt to achieve personalized humidity and thermal management.^[^ ^147^ ^]^ Heterogeneous humidity‐responsive textiles have also been developed. Wang et al. deposited hygroscopic microbial cells on both sides of a humidity‐inert material, creating a film with a heterogeneous multilayered structure (Figure 9d). The film exhibits a specific response to localized moisture gradients and was designed as ventilating bio‐flaps on sports apparel (Figure 9e) to enhance air convection.^[^ ^149^ ^]^

Moreover, textile materials (e.g., PE, PA) have also been utilized for humidity‐responsive actuators.^[^ ^135^ ^]^ Li et al. designed a multimodal adaptive wearable textile using a nylon‐silver (Ag)‐polystyrene‐block‐poly(ethylene‐ran‐butylene)‐block‐polystyrene heterostructure (Figure 9f). The presence of amide groups in nylon‐6 enables hygroscopic expansion and bimorph actuation. As shown in Figure 9g, smaller strain at the interface caused by depositing Ag layer leads to an increasing bending angle. This textile demonstrated enhanced evaporative heat dissipation through the bending of flaps in response to sweat vapor. As shown in Figure 9h, the device was attached to the backside of a commercially available T‐shirt (back). The flaps of the device are in the closed state at the beginning and fully opened When sweating. While the sweat vapor fully disappeared, the flaps returned to the initial closed state. Under the joint action of various heat dissipation pathways, the thermal comfort zone was expanded by 30.7%.^[^ ^150^ ^]^

Pinecone effect cooling textiles have gained commercial applications in sportswear. However, one major challenge lies in the 3D opening behavior of the membrane flap. This unique structure poses practical difficulties as accessories like backpacks and belts can obstruct the actuator, thereby limiting the wider application of pinecone effect cooling textiles.

### 6.2 Fiber/Yarn‐Based Responsive Cooling Textiles

**Figure 10:**
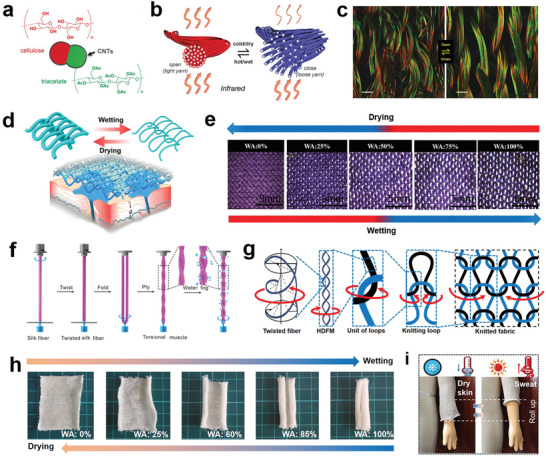
Dynamic responsive cooling textiles based on fiber/yarn structures. a) A metafiber design based on CNT‐coated triacetate‐cellulose side‐by‐side bimorph fibers. b) Design principles of an infrared gating yarn. The yarn is fluffy with large distances between the fibers. When hot and wet, the yarn collapses into a tight bundle. c) Confocal fluorescent microscopy images showing the knitted fabric in the closed state (low humidity) and the open state (high humidity). Reproduced with permission.^[^ ^151^ ^]^ Copyright 2019, The American Association for the Advancement of Science. d) Schematic of the reversible loop structure change during wetting and drying of the moisture‐responsive yarns. Reproduced with permission.^[^ ^152^ ^]^ Copyright 2019, Springer Nature. e) Light microscopy images of pore opening of knitted woolen fabric with increasing water absorption. Reproduced with permission.^[^ ^153^ ^]^ Copyright 2020, John Wiley & Sons. f) Schematic illustration of the fabrication of a two‐ply, torque‐balanced silk torsional muscle. Reproduced with permission.^[^ ^156^ ^]^ Copyright 2019, John Wiley & Sons. g) Hierarchical understanding of the torsional actuation of the twisted fiber. Red arrows indicate the rotation direction under humidity stimulation. h) Pictures of shape changes of the knitted fabric muscle upon water absorption. i) Pictures showing the thermal management effect of the sleeve of a garment in a dry skin state (left) and a sweat‐induced roll‐up state (right). Reproduced with permission.^[^ ^155^ ^]^ Copyright 2021, American Chemical Society.

Dynamic responsive cooling textiles that exhibit deformation in one or two dimensions (e.g., the fiber/yarn, or within the plane of the textile) offer significant advancements in terms of ergonomics. One effective strategy is the development of fibers/yarns with variable diameters based on water content, which can be woven into fabrics. The reduction in fiber/yarn diameter results in an increase in the pore area of the fabric, facilitating heat exchange between the microclimate and the environment. Zhang et al. developed bimorph fibers consisting of hydrophobic triacetate and hydrophilic cellulose, with CNTs coated on the fibers (**Figure** **10**a). When exposed to moisture, the meta‐elements on neighboring fibers come closer together, inducing resonant electromagnetic coupling. This coupling effect alters the emissivity of the textile, enhancing the thermal radiation from the human body (Figure 10b,c). In addition, the expansion of pores facilitates conventional heat exchange mechanisms such as convection, conduction, and evaporation.^[^ ^151^ ^]^ Fu et al. designed a knitted fabric with a bilayer structure incorporating moisture‐responsive yarns (consisting of cellulose and tri‐acetate components) outside and polyethylene terephthalate yarns inside. The moisture‐responsive yarns absorb sweat, causing a transition from a loose to a dense structure (Figure 10d). This design contributes to an increased porous area ranging from 10 to 47%, and an enhanced heat flux from 74.4 to 152.3 W cm^−2^.^[^ ^152^ ^]^ Hu et al. proved that wool yarn exhibits two‐way shape‐memory behavior in response to water. Water absorption by the wool fibers increases the yarn length and decreases the yarn diameter. The actuation of the woolen fabric under water stimulation enables pore switching (Figure 10e), allowing for thermoregulation in sweat‐inducing environments.^[^ ^153^ ^]^

Another strategy for achieving responsive cooling is the development of fiber‐based artificial muscles with excellent actuation performance.^[^ ^154^ ^]^ These artificial muscles, based on twisting and coiling techniques, have demonstrated significant torsional and tensile actuation in response to moisture stimulus, leveraging the mechanism of fiber volume expansion. Fabric muscles have the potential to transform the 1D rotation of yarn muscles into 2D rolling. It was because the bending energy from the natural edge curling of a single textile due to yarn curvatures is much lower than the bending energy derived from the twist and torsion of fibers and yarn muscles.^[^ ^155^ ^]^ Jia et al. fabricated the torsional silk muscles by twisting and folding silk fibers. As shown in Figure 10f, each individual fiber had a left‐handed twist (S twist), while the folded fiber had a right‐handed twist (Z twist). When absorbed water, the individual fiber with S twist will untwist due to the volume expansion, generating a torque and increasing the plying in the Z direction (opposite to the fiber twist). Water desorption reverses these processes. Smart textiles woven from silk fiber muscles can respond to humidity changes in the environment.^[^ ^156^ ^]^ Peng et al. constructed humidity‐driven yarn artificial muscles with a recorded torsional stroke of 1752° cm^−1^ and a maximum rotation speed of up to 2100 rpm. These muscles were further woven into large‐sized fabric muscles through textile technologies. In a wet state, the torsional energy stored in the twisted fibers is transferred to the yarn muscle, inducing a torsional stroke. This stroke creates unbalanced out‐of‐plane stress in the knit unit structure, leading to shape changes in the knitted fabric (Figure 10g,h). Upon drying, the yarn muscles reverse the torsional stroke, releasing the stress imposed on the knit unit structure and allowing the fabric to recover. When absorbing water, the knitted fabric undergoes self‐adaptive shape changes and can be applied as the sleeve of a garment. As illustrated in Figure 10i, when the human skin is sweating, the sleeve rolls up, enhancing sweat evaporation and heat loss, thus cooling down the body.^[^ ^155^ ^]^

In practical applications involving smart materials, several critical challenges remain. The responsive performance tends to degrade as the scale increases from the micro to the macro level. Consequently, addressing issues related to the speed and magnitude of stimulus response becomes a formidable task. Innovative textiles offer promising avenues for enhancing energy efficiency and represent intriguing alternatives for effectively managing human thermal comfort.

## 7 Conclusion and Perspective

**Figure 11:**
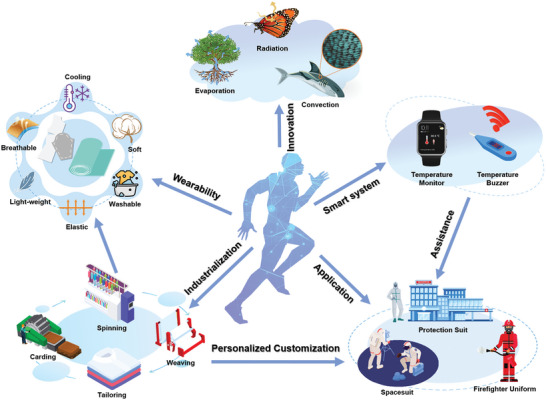
Challenges and perspectives for cooling textiles of next generation.

High‐temperature environments have detrimental effects on physical health and productivity, creating a strong demand for personal cooling solutions. This comprehensive review aims to provide an overview of the latest research findings and developments in the field of textile‐based personal cooling systems. By incorporating thermal management properties, textiles based on fibrous membranes, fibers/yarns, and fabric structures enable localized cooling, surpassing spatial limitations and better catering to individual needs. **Table** **1** summarizes the studies of five main types of cooling textiles including design principles, mechanisms, cooling effect, and limitations, offering theoretical guidance and new insights for further research in this area. Moreover, the use of cooling textiles as an alternative to traditional air conditioning can significantly reduce energy consumption and environmental pollution. Despite the significant progress, the current development and applications of cooling textiles still have some deficiencies. For promoting practical applications, future cooling textiles can develop in five main aspects, namely, innovative approaches and multidisciplinary integration, wearability, industrialization, personalized customization for specific applications, and smart personal thermal management system (**Figure** **11**).

1. Innovative approaches and multidisciplinary integration. To meet diverse environmental demands, it is desired to explore cooling textiles with innovative structures and functionalities. Integrating well‐established spinning and weaving techniques with knowledge from diverse fields such as materials science, thermophysics, physiology, psychology, electronics, and other relevant disciplines can significantly advance cooling textile technology. Furthermore, biomimetic research can be conducted to draw inspiration from nature and develop more innovative and practical personal cooling textiles.
2. Wearability of cooling textiles. Current cooling textiles predominantly rely on rigid electronic components, which are incompatible with soft garments and uncomfortable to wear. Therefore, a crucial aspect of cooling textiles is to ensure not only high cooling efficiency and temperature reduction but also good wearability performance, encompassing softness, breathability, washability, high strength, lightweight, and safety. It's a promising strategy to incorporate cooling functionality into fibers, yarns, and fabrics, thus offering both functionality and comfort.^[^ ^157^ ^]^
3. Industrial production of cooling textiles. Much of the research on cooling textiles remains in the theoretical and laboratory stages. Complex manufacturing processes and high production costs pose major obstacles to industrialization and commercialization. To expedite market adoption, it is vital to explore novel approaches that simplify manufacturing procedures, reduce costs, and integrate multiple functionalities into cooling textiles while maintaining high cooling efficiency.
4. Personalized customization for specific applications. Research on cooling textiles should become more practical and tailored to specific application scenarios and industries. For instance, the development of intelligent and high‐performance cooling textiles can expand their use in fields like firefighting and aerospace, extending their temperature regulation capabilities.
5. Establishing smart personal thermal management systems. Cooling textiles have the potential to evolve into multifunctional fabrics capable of regulating thermal comfort, monitoring health, enabling sensory interactions, and generating thermoelectric power. Integrating cooling textiles with sensing technology and flexible electronic devices to establish the smart system can achieve these functions and better align with the diverse needs of various physical activities and intelligent healthcare services. For instance, these textiles can employ sensing technology to gather and analyze data concerning human body temperature and thermal‐moisture comfort, subsequently providing feedback through autonomous thermoregulating of the textile.^[^ ^158^, ^159^, ^160^, ^161^, ^162^, ^163^ ^]^

**Table 1:** Summarization of mechanisms, cooling effect, and limitations of cooling textiles.

Category	Design principle	Heat transfer mode	Equation	Material	Cooling effect	Limitation
Device cooling textile	Air ventilation	Convection	*P_conv_* = *K_conv_* (*T_s_* − *T_e_*)	Garments with fans.	High airflow rate and high cooling efficiency.	Properties of rigid, bulk, uncomfortable. Needs a continuous power supply. High cost and low energy efficiency. Liquid cooling and thermoelectric devices need tightly attached to the human skin.
	Liquid cooling	Conduction	$\;P_{cond}=\frac{K_{cond}}{t_{t}}\;\left(T_{s}-T_{e}\right)$	Garments integrated with tubes circulating cooling liquid, such as water, and ethylene glycol.	High heat loss for the body.	
	Thermoelectric cooling			Sb_2_Te_3_, Bi_2_Te_3_, etc.	High cooling power, long effective cooling time.	
Radiative cooling textile	MIR transparent radiation; solar irradiance reflectance.	Transmitted radiation, reflected solar irradiance	$\;P_{rad}=\;\varepsilon \sigma \left(T_{s}^{4}-T_{e}^{4}\right)$	PE, PA, ZnO, etc.	High MIR transmittance, high solar reflectance, and skin temperature drop.	The risk of privacy disclosure under infrared cameras.
	MIR emissive radiation; solar irradiance reflectance.	Emitted radiation, reflected solar irradiance		PVDF, HFP, silk, TiO_2_, SiO_2_, etc.	High MIR emittance, high solar reflectance, and skin temperature drop.	Limited thermoregulation capacity and restricted by heat transfer coefficient.
Thermal conductive cooling textile	High crystallinity and orientation	Conduction	$\;P_{cond}=\frac{K_{cond}}{t_{t}}\;\left(T_{s}-T_{e}\right)$	UHMW‐PE fibers	High thermal conductivity and skin temperature drop.	The improvement on crystallinity and orientation of fibers is limited.
	High thermal conductivity			Graphene, CNT, BN, metal, etc.		Potential health risks of nanomaterials.
Evaporative cooling textile	Moisture sorption	Evaporation	*P_evap_* = *H_ev_* θ(χ_*s*_ − χ)	Hydrophilic polymers, Hydrophobic polymers.	Good moisture absorption ability and low HI.	Limited cooling effect after reaching saturation moisture absorption.
	Directional water transport				Excellent wicking capability and fast sweat evaporation rate.	Hard for industrialization on complicated structural engineering.
Dynamic responsive cooling textile	Opening and closing of membrane flaps	Radiation, convection, evaporation	P = P_rad_ + P_conv_ + P_evap_	Nafion, hydrophilic‐hydrophobic bilayer.	Enhanced ventilation, high MIR emittance, rapid sweat evaporation and heat release.	The 3D opening behavior of membrane flaps limited the application of accessories like backpacks and belts.
	Deformation of fiber/yarn			Shape‐memory yarns, artificial muscles.		Small deformation, and slow response.

Overall, the future of cooling textiles holds great promise, and continued research and development in this field will unlock new and exciting possibilities.

## Conflict of Interest

The authors declare no conflict of interest.
